# Osteo-Rheumatology: a new discipline?

**DOI:** 10.1186/ar3708

**Published:** 2012-03-08

**Authors:** Gerolamo Bianchi, Luigi Sinigaglia

**Affiliations:** 1 Division of Rheumatology, ASL3 Genovese, Genoa, Italy; 2 Division of Rheumatology, G. Pini Institute, Milan, Italy

## 

The "Bone Involvement in Arthritis" International Meeting was first held in Venice, in 2004, with the objective of bringing together distinguished international experts in the fields of bone metabolism and rheumatic diseases to discuss emerging knowledge regarding the interplay between rheumatic diseases and the bone tissue. The growing interest and the continuous progresses on the topic led to the organization of six other meetings, which included several different established clinicians and/or researchers.

Since its origin, the meeting focused on the interactions between the bone tissue, the immune system and the cartilage in osteoarthritis, rheumatoid arthritis and spondyloarthropathies, always considering the two sides of the coin: the basic and clinical. In past years, as well as this year, specific sessions were dedicated to osteoarthritis (OA), rheumatoid arthritis (RA) and ankylosing spondylitis, respectively, with a starting session considering the general aspects of the topic. In this context, the bone damage as an early manifestation of arthritides, the systemic skeletal involvement in RA, the crucial role played by subchondral bone in the pathogenesis and progression of OA, the pathophysiology of glucocorticoids damage on the bone tissue, and the potential beneficial effects of newly approved agents such as bisphosphonates and biologics, but not only, were discussed.

In 2011, the meeting has been named, for its first time, "Osteo-Rheumatology", thus implying the necessity of giving a more clear definition to topics presented and the issues raised. Even this year, experts in bone and rheumatic diseases interacted to improve our knowledge regarding the bone involvement in arthritis and to raise issues to be addressed in the future.

